# Multicentric assessment of the efficacy and tolerability of dihydroartemisinin-piperaquine compared to artemether-lumefantrine in the treatment of uncomplicated *Plasmodium falciparum *malaria in sub-Saharan Africa

**DOI:** 10.1186/1475-2875-10-198

**Published:** 2011-07-20

**Authors:** William Yavo, Babacar Faye, Thomas Kuete, Vincent Djohan, Serge A Oga, Richard R Kassi, Mariama Diatta, Moor V Ama, Roger Tine, Jean-Louis Ndiaye, Jean-Bedel Evi, Albert Same-Ekobo, Oumar Faye, Moussa Koné

**Affiliations:** 1 Department of Parasitology and Mycology, Faculty of Pharmaceutical and Biological Sciences, Abidjan, Côte d'Ivoire; 2 Parasitology and Mycology Laboratory, Faculty of Medecine, UCAD, Dakar, Sénégal; 3 Parasitology Laboratory, Faculty of Medecine and Pharmaceutical Sciences of Douala, Yaoundé, Cameroon; 4 Departement of Biostatistic and Public Health, Faculty of Pharmaceutical and Biological Sciences, Abidjan, Côte d'Ivoire; 5 Projet RETROCI, Abidjan, Côte d'Ivoire; 6 Malaria Research and Control Center, National Institute of Public Health, Abidjan, Côte d'Ivoire; 7 University Hospital Center, Yaoundé, Cameroon

## Abstract

**Background:**

The choice of appropriate artemisinin-based combination therapy depends on several factors (cost, efficacy, safety, reinfection rate and simplicity of administration). To assess whether the combination dihydroartemisinin-piperaquine (DP) could be an alternative to artemether-lumefantrine (AL), the efficacy and the tolerability of the two products for the treatment of uncomplicated falciparum malaria in sub-Saharan Africa have been compared.

**Methods:**

A multicentric open randomized controlled clinical trial of three-day treatment of DP against AL for the treatment of two parallel groups of patients aged two years and above and suffering from uncomplicated falciparum malaria was carried out in Cameroon, Côte d'Ivoire and Senegal. Within each group, patients were randomly assigned supervised treatment. DP was given once a day for three days and AL twice a day for three days. Follow-up visits were performed on day 1 to 4 and on day 7, 14, 21, 28 to evaluate clinical and parasitological results. The primary endpoint was the recovery rate by day 28.

**Results:**

Of 384 patients enrolled, 197 were assigned DP and 187 AL. The recovery rates adjusted by genotyping, 99.5% in the DP group and 98.9% in the AL group, were not statistically different (p = 0.538). No Early Therapeutic Failure (ETF) was observed. At day 28, two patients in the DP group and five in AL group had recurrent parasitaemia with *Plasmodium falciparum*. In the DP group, after PCR genotyping, one of the two recurrences was classified as a new infection and the other as recrudescence. In AL group, two recurrences were classified after correction by PCR as recrudescence. All cases of recrudescence were classified as Late Parasitological Failure (LPF). In each group, a rapid recovery from fever and parasitaemia was noticed. More than 90% of patients did no longer present fever or parasitaemia 48 hours after treatment. Both drugs were well tolerated. Indeed, no serious adverse events were reported during the follow-up period. Most of the adverse events which developed were moderate and did not result in the treatment being stopped in either treatment group.

**Conclusions:**

Dihydroartemisinin-piperaquine was as effective and well-tolerated as artemether-lumefantrine in the treatment of uncomplicated falciparum malaria. In addition, dihydroartemisinin-piperaquine, a single daily dose, could be an advantage over artemether-lumefantrine in Africa because of better treatment observance.

## Background

Malaria caused by *Plasmodium falciparum *is a serious concern for public health and development in Africa. The most part of the continent is facing increasing resistance of this parasite to chloroquine and sulphadoxine-pyrimethamine, the widely available and cheap anti-malarial drugs. In order to overcome this resistance problem, several African countries have recently adopted artemisinin-based combination therapy (ACT) as first-line treatment for uncomplicated malaria [1,2]. The artemether-lumefantrine (AL) combination proved to be highly effective and well-tolerated in several studies in Africa [3-5]. However, a twice daily dose schedule of AL and its need to be administered together with a fat-rich meal [6] are a disadvantage.

Dihydroartemisinin-piperaquine (DP) is a new ACT administered as single daily dose that has proved to be well tolerated and highly effective against uncomplicated falciparum malaria in southeast Asia [7-12] and in eastern Africa [13,14]. However, no clinical trial concerning this anti-malarial drug has yet been conducted in western and central Africa. Therefore, a randomized non-inferiority open trial has been carried out to compare efficacy and tolerability of DP to that of AL, the reference ACT, in the treatment of uncomplicated falciparum malaria in Cameroon, Côte d'Ivoire and Sénégal. The hypothesis was that DP is as efficacious and safe as AL.

## Methods

### Study site and population

This study was carried out from November 2006 to May 2008 in three sub-Saharan countries: Senegal and Côte d'Ivoire are in western Africa, Cameroon is in central Africa. In Cameroon, the study took place at the University Hospital Centre. In Côte d'Ivoire, it took place at the health care centre of Bocabo, which is located in Abobo in the north of Abidjan (the economic capital). In Senegal, the assessment took place at the health care centre of Darou Marnane in the sanitary district of Touba, which is located in the centre of the country at 200 km of Dakar.

The study population consisted of outpatients visiting health care centres for uncomplicated falciparum malaria-like symptoms. Patients were enrolled if they meet the following selection criteria: 1) at least two years old; 2) fever with axillary temperature > or = 37.5°C; 3) *P. falciparum *mono-infection with a parasitaemia from 2,000 to 200,000/μl of blood, in Cameroon and Côte d'Ivoire, and from 1,000 to 100,000/μl of blood in Sénégal; 4) provision of the written and informed consent by the patient or his legal guardian for children; 5) no history of previous serious side-effects to the drugs used in the trial; 6) no evidence of a concomitant febrile illness; 7) no danger signs or evidence of severe malaria; 8) no treatment with 4-amino quinoleines, sulphadoxine-pyrimethamine, mefloquine or halofantrine in the previous seven days, or with quinine, artemisinin or cyclins in the previous three days, 9) no pregnancy or nursing; 10) no ongoing anti-malarial treatment.

The number of patients to be enrolled was determined by Epi Info 2000 software. The estimated expected recovery rate with AL was 98%, with a maximum acceptable difference of 5% to conclude that DP was non-inferior and a power of 85%. The minimum number of people to be included in each arm was calculated from these assumptions to be 180 patients. Assuming a 5% loss to follow up the overall final target sample size of 380 participants was estimated.

### Study design and treatment

This study was a randomized, controlled and open therapeutical trial of DP against AL as the reference treatment. The three criteria of judgment were clinical efficacy, biological efficacy and tolerance. The study protocol was first approved in each country by the National Ethical Committee according to protocols and standards operating procedures of Good Clinical Practices of the ICH harmonized Triplicate Guide Lines for Good Clinical Practice made in 1996 and the Helsinki Declaration on human being research. This approval was critical for the study start.

For each patient who met inclusion criteria, the protocol was read and explained to him/her or the legal guardian (for children) who in case of acceptance had to sign the written informed sheet afterward to authorize recruitment of the child in the study. The patient or guardian was given a copy of the informed consent and patient information sheet. For patients who gave their consent, baseline examinations and laboratory investigations were carried immediately free of charge. Those of patients who met inclusion criteria at baseline (day 1) were randomly assigned to one of the two treatment groups following a randomization list. In each study site computer generated randomization codes were prepared by an independent individual. These codes were enclosed in sequentially numbered opaque sealed envelopes, each of which contained the treatment allocation. The envelopes were assigned in sequential order to participants after inclusion.

Treatments in the two groups were allocated according to body weight. First patient group was allocated dihydroartemisinin-piperaquine (DP) (Duocotecxin^® ^) (Beijing Holley-Cotec Pharmaceutical Co. Ltd, China) once daily for three consecutive days. Each tablet of DP contains 40 mg of dihydroartemisinin and 320 mg of piperaquine. DP treatments varied as follows: patients between 5-9 kg received half a tablet per dose, those between 10-14 kg 3/4 tablet, 15-19 kg 1 tablet per dose, 20-24 kg 1 tablet + 1/4 per dose, 25-29 kg 1 tablet + 1/2 per dose, 30-34 kg 1 tablet + 3/4 per dose, 35-39 kg 2 tablets per dose, 40-44 kg 2 tablets + 1/4 per dose, 45-49 kg 2 tablets + 1/2 per dose and patients ≥ 50 kg 3 tablets per dose. The second patient group was allocated artemether-lumefantrine (AL) (Coartem^® ^) tablets (Novartis Pharma, Switzerland), each tablet containing 20 mg artemether and 120 mg lumefantrine. Patients received treatment dose according to the following scheme: i.e. patients between 5-14 kg received one tablet per dose, those between 15-24 kg two tablets, those between 25-34 kg received three tablets per dose and those with body weight ≥ to 35 kg received four tablets per dose. Doses were given at T0h, T8h, T24h, T36h, T48h and T60h. All treatments were given under direct supervision of the study co-investigators. If the patient vomited within thirty minutes after drug administration, the whole dose was re-administered. If the vomiting persisted, the patient was excluded from the study and referred to the health centre doctor for management according to the current national policy. The dose could not be administered again if vomiting occurred more than 60 minutes after administration.

After inclusion, the patient had to come to follow-up visits during which clinical and physical examinations as well as laboratory investigations were carried out by the physician and laboratory technicians.

### Efficacy and tolerance

Clinical and biological efficacies were assessed using the WHO in vivo test with a follow-up period of 28 days [14,15]. At enrollment (day 1) as well as follow-up visits, a full clinical and physical examination was performed; data were recorded in a case report form. Laboratory investigations for *P. falciparum *stages and loads were also carried out at baseline and follow-up visits by means of finger pricking for thin and thick smears. Follow-up visits were on day 2, day 3, day 4, day 7, day 14, day 21 and day 28 after the first anti-malarial drug had been swallowed. Finger prick blood samples were collected for microscopy. Blood samples were also collected for *P. falciparum *molecular biology analysis at baseline and then after day 7 in case of positive parasitaemia to check if parasitaemia recurred in patients. Response to treatment was measured and defined according to WHO guidelines [15]. Other blood samples were collected by venipuncture at baseline and day 4 for the measurement of haemoglobin levels and biochemical parameters, such as bilirubin, creatinin and transaminases (AST, ALT). Patients developing danger signs during follow-up visits were withdrawn from the study, referred to the appropriate hospital ward for care and medication. Other patients showing complications or treatment failure were also referred for appropriate care and medication but remained in the study for the follow-up. Adverse events (AE) were recorded on the case report forms and their gravity was graded as mild, moderate or severe. An AE was defined as an unfavourable and unintended symptom, sign or disease.

### Parasite clearance and gametocyte dynamics

*Plasmodium falciparum *parasite clearance was assessed microscopically. For this, Giemsa-stained thick and thin blood smears were prepared according to WHO guidelines [15]. Two independent, experienced microscopists, examined the smears for the presence and quantification of parasites species.

### Genotyping

In order to distinguish re-infection (RI) from recrudescence (RE), merozoite surface protein 1 and 2 (*msp1 *and *msp2*) genotyping was performed as described by Faye [16] on dried blood spots collected at the patient enrollment (day 1) and at the time when parasitaemia reccurrence was noticed. Blood spots were collected on Whatman filter paper (Whatman International Ltd. Maidstone, UK) and air-dried at room temperature for PCR analysis. DNAwas extracted using the methanol method [17]. Molecular biology analysis was performed at the Parasitology Laboratory of Cheikh Anta Diop University (Dakar, Sénégal).

### End points

The primary end point was the recovery rate by 28, defined as the percentage of patients who had an adequate clinical and parasitological response (ACPR) after follow-up for 28 days. Efficacy was evaluated using a modified intention to treat analysis, which included the 379 patients, randomized and not lost to follow-up. The secondary end points were the incidence of early clinical failure (ECF), late parasitological failure (LPF), late clinical failure (LCF), change in gametocyte carrier status, fever and parasites clearance and adverse clinical and laboratory events.

### Statistical analysis

All data were recorded and checked using Epi data version 3.1 and analysed with SPSS for windows (version 12.0). Patient's characteristics of the two groups at inclusion were compared using Pearson's Chi-2 test, independent samples t test or Mann-Whitney test. The modified intention to treat analysis was performed using Kaplan-Meier survival analysis with log rank testing the treatment failures distribution function. The cases of protocol violation and withdrawn consent were censored at the time they left the study. The distributions of fever and parasite clearance were compared using Pearson's Chi-2 test. Differences of haemoglobin and biochemical parameters values within individuals between D1 and D4 were computed. Changes in haemoglobin concentrations and in biochemical parameters were compared using the paired t test. The level of significance for statistical tests was set at 0.05.

## Results

### Global distribution and baseline characteristics of patients in the study

A total of 384 patients were included in the study. 197 patients were randomized to DP and 187 to AL. In the DP group, six patients were excluded after enrollment. Of these, four patients were lost during follow-up and one case of protocol violation (self-medication with another anti-malarial drug) and one case of withdrawal of consent were noticed. In the AL group, four patients were excluded during the follow-up: one patient was lost sight. There were one case of protocol violation and two cases of withdrawal of the consent.

**Figure 1:**
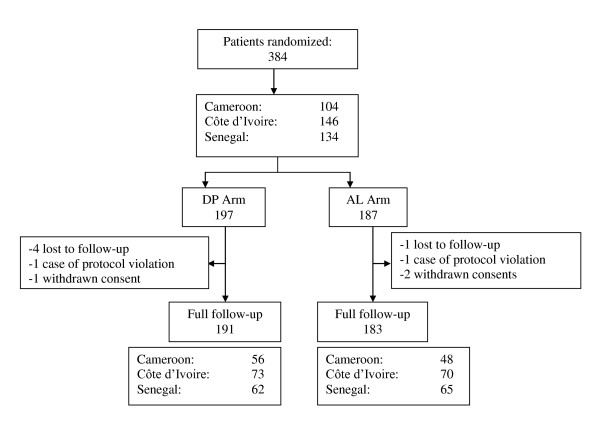
**Global distribution of patients in the study**.

Finally, 191 patients and 183 patients were successfully followed up, respectively in DP and AL groups (Figure 1). Baseline characteristics of patients receiving either AL or DP are presented in Table 1 and Table 2. There were no statistically significant differences in distribution by sex or age band. Average temperatures in the two treatment groups were not statistically different. There were also no statistically significant differences between any of the laboratory parameters. Both groups were, therefore, considered statistically equivalent at inclusion.

**Table 1:** Comparison of the two treatment groups at inclusion

	DP	AL	p*
	
Numbers	197	187	
**Sex**			
**M, n *(%)***	97 *(49.2)*	93 *(47.2)*	0.923**
**F, n *(%)***	100 (*50.8)*	94 *(52.8)*	

**Mean age (SD) years**	15.64 (*14.22*)	13.48 (*12.64*)	*0.117*
**[2 - 5[, n *(%)***	34 *(17.3)*	44 *(23.7)*	
**[5 - 15[, n *(%)***	92 *(46.7)*	83 *(44.6)*	
**[15-77], n *(%)***	71 *(36.0)*	59 *(31.7)*	

**Mean temperature (SD) °C**	38.42 (0.64)	38.43 (0.73)	*0.887*
**[37.5 - 38.5[, n *(%)***	114 *(57.9)*	113 *(60.4)*	
**[38.5 - 41.0], n *(%)***	83 *(42.1)*	74 *(39.6)*	

**Median parasitaemia tpz/μl**	10840	16000	*0.546*§

**Min - Max**	1000 - 200000	1000 - 200000	

**Gametocyte carrier rate, n *(%)***	8 *(4.1)*	6 *(3.2)*	0.649**

**Mean AST (SD) IU/l**	26.64 (15.53)	31.1 (29.08)	*0.065*

**Mean ALT (SD) IU/l**	18.71 (12.71)	24.06 (36.63)	*0.061*

**Mean creatinin (SD) mg/l**	6.79 (2.41)	6.76 (2.47)	*0.915*

**Mean bilirubin (SD) mg/l**	10.43 (7.21)	11.4 (8.65)	*0.236*

**Mean haemoglobin (SD) g/dl**	10.79 (2.06)	10.67 (2.12)	*0.575*

*Independent samples t test **Pearson's Chi-2 test §Mann-Whitney test

**Table 2:** Clinical signs of patients at inclusion

	DP (n, %)	AL (n, %)	Total n (%)
**Fever**	197 (100%)	187 (100%)	384 (100)
**Headache**	167 (84.8%)	153 (81.8%)	320 (83.3)
**Asthenia**	153 (81.8%)	106 (56.7%)	223 (58.1)
**Anorexia**	103 (52.3%)	95 (50.8%)	198 (51.6)
**Shiver**	93 (47.2%)	80 (42.8%)	173 (45.1)
**Joint pain**	77 (39.1%)	75 (40.1%)	152 (39.6)
**Abdominal pain**	35 (17.8%)	43 (23%)	78 (20.3)
**Insomnia**	18 (9.1%)	23 (12.3%)	41 (10.7)
**Dizziness**	18 (9.1%)	13 (6.9%)	31 (8.1)

### Clinical and parasitological outcomes

Kaplan-Meier estimates of recovery rates unadjusted by genotyping were 99.0% in the DP group and 97.3% in the AL group. There was no statistical difference between the two groups (p = 0.230). These recovery rates adjusted by genotyping, 99.5% in the DP group and 98.9% in the AL group, were not statistically different (p = 0.538). There was no significant study site effect on these estimates.

No Early Therapeutic Failure (ETF) was observed. At day 28, 2 patients in the DP group and 5 in AL group had recurrent parasitaemia with *P falciparum*. In the DP group, after PCR genotyping, one of the two recurrences was classified as a new infection and the other as recrudescence. In AL group, two recurrences were classified after correction by PCR as recrudescence. All cases of recrudescence were classified as Late Parasitological Failure (LPF).

**Figure 2:**
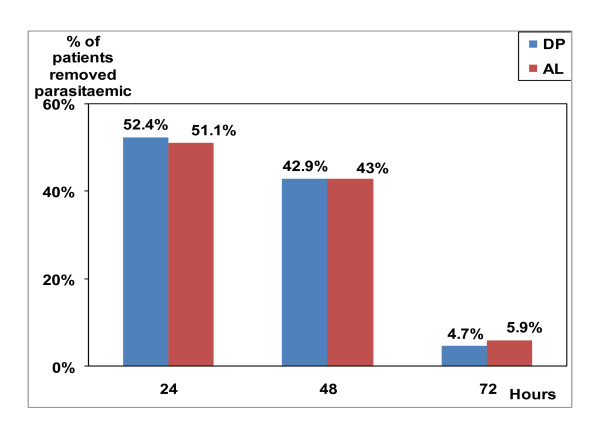
**Parasite clearance**.

**Figure 3:**
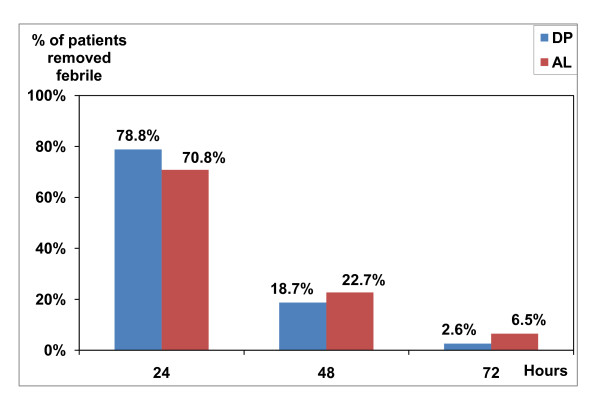
**Fever clearance**.

In the two groups, almost 94% of patients had cleared parasitaemia within 48 hours after enrollment; there was no statistical difference between the two groups (p = 0.866) (Figure 2). Fever clearance was equally high: at day 3 almost all patients were apyretic, with no difference between the 2 groups (p = 0.94) (Figure 3). At inclusion, there were only eight gametocyte carriers in the DP group and six in the AL group. The number of gametocyte carriers decreased to one on day 3 in the DP group and to two in the AL group. From day 4, there were no more gametocyte carriers in both groups.

### Adverse events

Among the 384 patients followed, 35 (23 in DP group and 12 in AL group) had adverse events (9.1%). There was no severe adverse event in the two groups and no treatment was interrupted due to adverse events. Most of adverse events were of moderate severity. The most commonly reported adverse events in both treatment groups were abdominal pain, dizziness, diarrhoea, vomiting, pruritus and nausea (Table 3). Adverse events were not significantly different between the two treatment groups.

**Table 3:** Adverse events recorded during the study

Adverse events	DP (n, %)	AL (n, %)	Total (n, %)
**Abdominal pain**	9 (4.6)	9 (4.8)	18 (4.7)
**Dizziness**	5 (2.5)	2 (1.1)	7 (1.8)
**Diarrhoea**	4 (2)	2 (1.1)	6 (1.6)
**Vomiting**	4 (2)	0 (0)	4 (1)
**Pruritus**	3 (1.5)	1 (0.5)	4 (1)
**Nausea**	2 (1)	2 (1.1)	4 (1)
**Face oedema**	1 (0.5)	0 (0)	1 (0.3)
**Sleepiness**	1 (0.5)	0 (0)	1 (0.3)
**Insomnia**	0 (0)	1 (0.5)	1 (0.3)

A decrease of haemoglobin values was observed from the beginning of treatment to day 4. This decrease was more important in AL group (0.36 g/dl) than DP group (0.14/g/dl). The decrease was significant in AL group (p = 0.001) but not in DP group (p = 0.221). In DP group from the beginning of the treatment to day 4, there was a decrease of the mean of AST and a small increase of ALT mean, while in the AL group, AST and ALT means increased. However, these variations were not significantly different. The decrease of the mean of creatinin from the beginning of the treatment to day 4 was not significant in the DP group but was significant in the AL group. In the two groups, the bilirubin decrease was significant (Table 4).

**Table 4:** Evolution of biological parameters in the two groups

	DP				AL			
	
	D1	D4	D1-D4	p*	D1	D4	D1-D4	p*
**AST (IU/l)** **(SD)**	26.48 (15.17)	24.79 (12.10)	1.70 (17.51)	**0.190**	29.62 (26.14)	38.24 (131.0)	- 8.63 (132.6)	**0.389**
**ALT (IU/l)** **(SD)**	18.91 (12.71)	19.73 (12.91)	- 0.82 (13.77)	**0.418**	24.20 (37.46)	34.19 (138.47)	- 9.99 (140.5)	**0.347**
**Creatinin (mg/l)** **(SD)**	6.81 (2.46)	6.70 (2.41)	0.11 (1.52)	**0.317**	6.86 (2.45)	6.51 (2.51)	0.35 (1.9)	**0.001**
**Bilirubin (mg/l)** **(SD)**	10.49 (7.39)	6.31 (2.99)	4.18 (7.42)	**< 0.001**	11.19 (8.51)	6.27 (3.30)	4.92 (8.57)	**< 0.001**
**Haemoglobin (g/dl)** **(SD)**	10.80 (2.07)	10.66 (2.18)	0.14 (1.56)	**0.221**	10.75 (2.07)	10.39 (2.08)	0.36 (1.48)	**0.001**

*paired t test

## Discussion

In this study, the efficacy and tolerability of two ACT formulations have been compared in a randomized trial of patients with uncomplicated malaria and followed up for 28 days. Both treatments were highly efficient and well tolerated as previously described [1]. Moreover, several studies with a follow-up of 42 days have been carried out about the efficacy and tolerability of DP and AL and all showed very good results [18-21]. The rate of true recrudescence with *P. falciparum *was less than 5% in both treatment groups, and most *P. falciparum *recurrences were caused by reinfection. These outcomes are in accordance with previous efficacy studies of AL [13,23] and DP [7,9,11,24,25].

The fact that there was more reinfection than recrudescence shows that malaria transmission is very high in these areas (especially in Côte d'Ivoire and Cameroon). Thus, even if anti-malarial drugs are effective and well-tolerated, it is very important to continue to use prevention tools, such as long-lasting insecticide-treated nets to fight malaria.

The haemoglobin rate decrease was significant in the AL group, but not in the DP group. This result suggests that convalescence is obtained faster in the DP group. However, as these data were obtained only four days after enrollment, it would be more significant to assess the haemoglobin rate decrease over 28 days. As in several studies which reported good tolerance of DP [25,26] and AL [26], there were no serious adverse events during the follow-up in the assessment.

In conclusion, DP presented good and equivalent efficacy and tolerability profile as AL. Therefore, DP is a good alternative for the first-line treatment of uncomplicated *P. falciparum *malaria in endemic regions. Its once daily dose is even more a significant advantage because it could contribute to improve the patient's treatment compliance.

## List of abbreviations

DP: combination Dihydroartemisinin - Piperaquine phosphate; AL: Artemether - Lumefantrine.

## Competing interests

The authors declare that they have no competing interests.

## Authors' contributions

OF, SE, KM supervised the clinical studies. KM was the principal investigator. SAO, WY and KM analysed the data. All authors read and approved the final manuscript.
